# In-hospital major adverse cardiovascular events after primary percutaneous coronary intervention in patients with acute ST-segment elevation myocardial infarction: a retrospective study under the China chest pain center (standard center) treatment system

**DOI:** 10.1186/s12872-023-03214-x

**Published:** 2023-04-17

**Authors:** Luyao Huang, Jing Zhang, Qing Huang, Ruiqing Cui, Jian Chen

**Affiliations:** 1 Department of Cardiology, The Second People’s Hospital of Hefei, Hefei Hospital Affiliated to Anhui Medical University, Hefei, 230011 Anhui China; 2 The Fifth School of Clinical Medicine, Anhui Medical University, Hefei, 230032 Anhui China; 3 Department of Clinical Laboratory, The Second People’s Hospital of Hefei, Hefei Hospital Affiliated to Anhui Medical University, Hefei, 230011 Anhui China

**Keywords:** Chest pain center, ST-segment elevation myocardial infarction, Major adverse cardiovascular events, Percutaneous coronary intervention

## Abstract

**Background:**

Patients with acute ST-segment elevation myocardial infarction (STEMI) undergoing primary percutaneous coronary intervention (PCI) are at high risk of major adverse cardiovascular events (MACE) despite timely treatment. This study aimed to investigate the independent predictors and their predictive value of in-hospital MACE after primary PCI in patients with acute STEMI under the China chest pain center (standard center) treatment system.

**Methods:**

We performed a single-center, retrospective study of 151 patients with acute STEMI undergoing primary PCI. All patients were treated under the China chest pain center (standard center) treatment system. The data collected included general data, vital signs, auxiliary examination results, data related to interventional therapy, and various treatment delays. The primary endpoint was the in-hospital MACE defined as the composite of all-cause death, stroke, nonfatal recurrent myocardial infarction, new-onset heart failure, and malignant arrhythmias.

**Results:**

In-hospital MACE occurred in 71 of 151 patients with acute STEMI undergoing primary PCI. Logistic regression analysis showed that age, cardiac troponin I (cTnI), serum creatinine (sCr), multivessel coronary artery disease, and Killip class III/IV were risk factors for in-hospital MACE, whereas estimated glomerular filtration rate (eGFR), left ventricular ejection fraction (LVEF), systolic blood pressure (SBP), diastolic blood pressure (DBP), were protective factors, with eGFR, LVEF, cTnI, SBP, and Killip class III/IV being independent predictors of in-hospital MACE. The prediction model had good discrimination with an area under the curve = 0. 778 (95%CI: 0.690–0.865). Good calibration and clinical utility were observed through the calibration and decision curves, respectively.

**Conclusions:**

Our data suggest that eGFR, LVEF, cTnI, SBP, and Killip class III/IV independently predict in-hospital MACE after primary PCI in patients with acute STEMI, and the prediction model constructed based on the above factors could be useful for individual risk assessment and early management guidance.

## Background

Recent studies have highlighted the decrease in acute and long-term mortality after acute ST-segment elevation myocardial infarction (STEMI) [1–3]. Nevertheless, the mortality remains high. The morbidity and mortality in patients with acute STEMI have trended downward for decades in developed countries but have continued to rise in China [4]. Among patients admitted to the hospital for acute STEMI, there is a persistent gap between practice and the recommended medical care, and the outcomes have not improved significantly during the decade 2001–2011 [5].

To improve the healthcare quality of patients with acute myocardial infarction (AMI), the Chinese Society of Cardiology began accrediting chest pain centers (CPC) in 2013. After the American CPC and German chest pain unit, the China CPC certification is the third professional accreditation system. The China CPC accreditation is divided into standard and preliminary centers, certifying medical institutions with different capabilities. Standard center accreditation requires the ability to perform emergency percutaneous coronary intervention (PCI) around the clock, while the preliminary center accreditation focuses more on thrombolysis and referrals. Based on a large national registry dataset, CPC accreditation is associated with better in-hospital outcomes in patients with AMI, as evidenced by a reduced risk of major adverse cardiovascular events (MACE) and all-cause death [6]. However, despite timely treatment, some patients may have unsatisfactory clinical outcomes, that is, a high incidence of MACE after primary PCI [7].

The current studies on the occurrence of in-hospital MACE in patients with acute STEMI after primary PCI have focused on general clinical conditions, auxiliary examination results, and the effect of CPC accreditation, while there are few studies with a comprehensive analysis under the China CPC (standard center) treatment system. This study aims to investigate the independent predictors and their predictive value of in-hospital MACE after primary PCI in patients with acute STEMI under the China CPC (standard center) treatment system and to provide a clinical basis for the early prognosis of patients with acute STEMI and the optimization of the management of treatment delays in CPC.

## Methods

### Study subjects and groupings

**Fig. 1:**
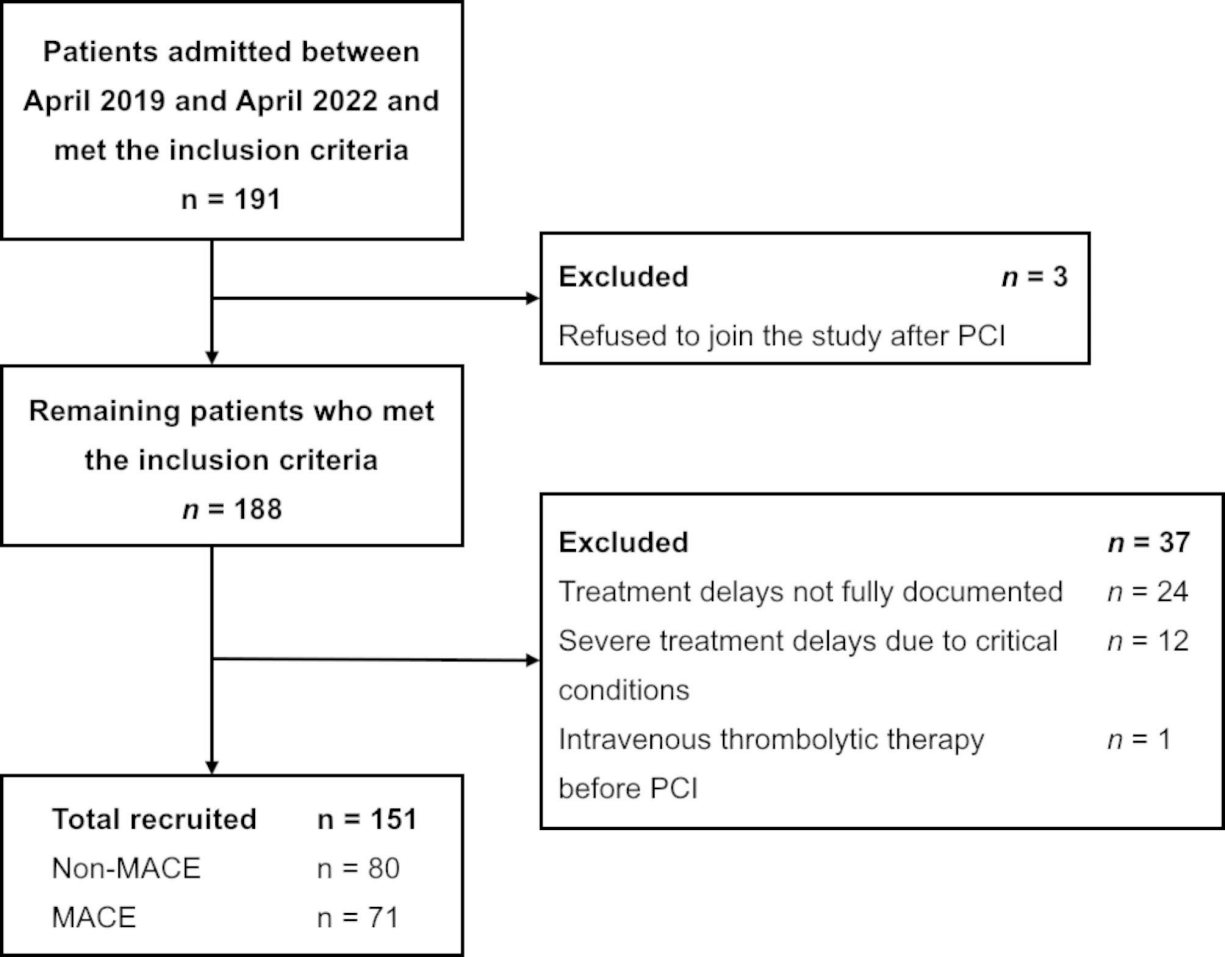
Study population flow chart

We conducted a retrospective, single-center observational study at the Second People’s Hospital of Hefei (Anhui Province, China), one of the standard centers under the China CPC network (Fig. 1). In this study, 151 patients (mean age 62 ± 14 years; 123 men and 28 women) admitted to the CPC between April 2019 and April 2022 with a definite diagnosis of acute STEMI and undergoing primary PCI were included. All patients were treated according to the China CPC (standard center) treatment procedure. A 12- or 18-lead electrocardiogram (ECG) was completed within 10 min after the first medical contact (FMC), and point-of-care testing (POCT) for cardiac troponin (cTn) was completed within 20 min. After activation of the cardiac catheterization laboratory (cath lab) following a diagnosis of acute STEMI, the patient went directly to the cardiac cath lab for PCI, with a single bypass cardiac care unit or double bypass emergency department and cardiac care unit. The inclusion criteria were as follows: (a) the diagnostic criteria met the “2019 Chinese Society of Cardiology (CSC) guidelines for the diagnosis and management of patients with ST-segment elevation myocardial infarction” [8]; (b) the patients met the primary PCI indications, and informed consent was signed; (c) aspirin 300 mg and a loading dose of ticagrelor 180 mg had been given to the patient before primary PCI; (d) only the culprit vessel was treated and successfully revascularized during primary PCI. The exclusion criteria were as follows: (a) patients whose treatment delays were not fully documented; (b) patients who suffered severe treatment delays due to hemodynamic instability or acute and critical conditions; and (c) patients treated with emergency intravenous thrombolytic therapy before PCI. The total cohort was divided into two groups, with the patients who did not experience in-hospital MACE in one group (non-MACE group, *n* = 80, 53.0%) and the patients who experienced in-hospital MACE in the other group (MACE group, *n* = 71, 47.0%). The study protocol and informed consent procedures were approved by the Ethics Committee of the Second People’s Hospital of Hefei.

### Data collection and outcome

Patient data were collected, including general data, vital signs, auxiliary examination results, treatment delays, and data related to interventional therapy. The general data included age, sex, and history of smoking. The patients’ past medical history included a history of hypertension and diabetes mellitus. The vital signs including the patient’s heart rate, systolic blood pressure (SBP), diastolic blood pressure (DBP), and Killip class were collected at the FMC. The auxiliary examination results included neutrophil count, platelet count, total cholesterol, triglycerides, high-density lipoprotein cholesterol (HDL-C), low-density lipoprotein cholesterol (LDL-C), random blood glucose, serum creatinine (sCr) (all collected within 24 h of admission), cardiac troponin I (cTnI) (collected once each in the outpatient clinic and 4–6 h after PCI, then took the higher value), left ventricular end-systolic dimension (LVESD), left ventricular end-diastolic dimension (LVEDD), left ventricular ejection fraction (LVEF) (all collected within 24 h of admission), and estimated glomerular filtration rate (eGFR) which was calculated using the CKD-EPI Creatinine Equation (2021) based on sCr. Data related to interventional therapy, which included the number of diseased coronaries (single/double/multiple), culprit vessels, and post-PCI TIMI flow grade, were obtained. The treatment delays included symptom-to-first medical contact (S-to-FMC) time, first medical contact-to-electrocardiogram (FMC-to-ECG) time, electrocardiogram-to-interpretation (ECG-to-I) time, cardiac troponin-to-result (cTn-to-R) time, cath lab activation time, door-to-balloon (D2B) time, and total ischemic time (TIT). The primary endpoint was in-hospital MACE.

### Definitions

In our study, MACE included all-cause death, stroke, nonfatal recurrent myocardial infarction, new-onset heart failure, and malignant arrhythmias. FMC was defined as the time between the initial assessment of the patient by a physician or other trained emergency medical services (EMS) personnel. S-to-FMC time was defined as the time from the onset of symptoms that compelled the patient to develop a desire to seek medical care to the time of the FMC. FMC-to-ECG time was defined as the time between the FMC and the performance of the first ECG. ECG-to-I time was defined as the time from the performance of the first ECG to the formal interpretation by a physician. cTn-to-R time was defined as the time from sampling to obtaining the results of POCT for cTn. Cath lab activation time was defined as the time between the decision to perform PCI and the arrival of the last interventionalist to the cardiac cath lab. D2B time was defined as the time from the patient’s arrival at the hospital door to the crossing of the catheter guidewire through the culprit lesion. TIT is defined as the time that elapsed from chest pain onset until the restoration of coronary blood flow.

### Statistical analysis

Continuous variables are presented as the mean ± standard deviation or median (interquartile range) depending on whether the variables had a normal distribution, and the variables were analyzed with Student’s t-test or the Mann‒Whitney U test. Categorical variables were expressed as numbers and percentages and were analyzed with the chi-squared test or Fisher’s exact test. Variables considered to be significant between the non-MACE and MACE groups or clinically relevant were entered into univariate logistic regression analyses, and backward stepwise multivariate logistic regression analysis (entry only if *P* < 0.05 and removal only if *P* > 0.10) was further applied to identify the independent predictors of in-hospital MACE. A nomogram was constructed based on the independent risk factors. We used the bootstrap method for internal validation, with resampling times B = 1000. The area under the receiver operating characteristic curve was applied to model discrimination evaluation. The calibration curve was applied to the model calibration assessment, and decision curve analysis was used to assess the clinical utility. All statistical analyses were two-sided, and *P* < 0.05 was considered statistically significant. Statistical analysis was performed using IBM SPSS Statistics 26.0 (IBM Corp., Armonk, NY, USA) and R software (version 4.2.2, www.R-project.org). The preliminary data were organized using Microsoft® Excel® 2019 (Microsoft Corporation, 2018).

## Results

### Baseline characteristics

The patients with in-hospital MACE had higher age, cTnI level, sCr level, and proportion of Killip class III/IV and lower SBP, DBP, and eGFR than those without in-hospital MACE (*P* < 0.05). The rest of the clinical conditions and auxiliary examination results were not significantly different (Table 1).

**Table 1:** Baseline characteristics

Variables	Non-MACE group	MACE group	*P*-value
	*n* = 80	*n* = 71	
Age, years	59.3 ± 14.8	65.8 ± 12.6	0.005*
Men, n (%)	67 (83.8)	56 (78.9)	0.442
Hypertension, n (%)	43 (53.8)	37 (52.1)	0.841
Diabetes, n (%)	25 (31.3)	20 (28.2)	0.680
Smoker, n (%)	39 (48.8)	35 (49.3)	0.947
Heart rate, bpm	76.0 (68.0, 87.0)	70.0 (58.0, 92.0)	0.097
SBP, mmHg	139.0 ± 27.9	126.1 ± 28.7	0.006*
DBP, mmHg	85.0 ± 20.4	76.9 ± 19.0	0.012*
Neutrophils, 10^9^/L	7.2 (5.8, 9.6)	7.7 (5.3, 9.9)	0.586
Platelet, 10^9^/L	201.7 ± 65.2	215.7 ± 66.0	0.193
Total cholesterol, mmol/L	4.63 ± 0.93	4.61 ± 1.10	0.904
Triglycerides, mmol/L	1.4 (1.0, 2.2)	1.3 (0.9, 2.1)	0.580
HDL-C, mmol/L	1.08 ± 0.22	1.09 ± 0.21	0.772
LDL-C, mmol/L	2.98 ± 0.76	2.89 ± 1.01	0.550
Blood glucose, mmol/L	7.0 (5.8, 9.2)	7.6 (6.1, 10.5)	0.229
sCr, mg/dL	0.8 (0.6, 0.9)	0.8 (0.7, 1.0)	0.012*
eGFR, mL/min/1.73m^2^	102.5 (89.8, 110.0)	91.0 (75.0, 104.0)	< 0.001*
cTnI, ng/mL	36.2 (14.7, 50.0)	50.0 (39.5, 50.0)	0.009*
Killip class III/IV, n (%)	16 (20.0)	36 (50.7)	< 0.001*

All data are described as number (percentage), mean ± standard deviation, or median (interquartile range), as appropriate. cTnI: cardiac troponin I; eGFR, estimated glomerular filtration rate; DBP, diastolic blood pressure; HDL-C, high-density lipoprotein cholesterol; LDL-C, low-density lipoprotein cholesterol; SBP, systolic blood pressure; sCr, serum creatinine

* Statistically significant

### Coronary angiography and echocardiography

The patients with in-hospital MACE had a higher frequency of multivessel coronary artery disease (MVCAD) and lower LVEF than the patients without in-hospital MACE (P < 0.05), and there were no significant differences in the distribution of culprit vessels, the proportion of post-PCI TIMI flow grade 0 to 2, LVESD, or LVEDD (Table 2).

**Table 2:** Coronary angiography and echocardiography

Variables	Non-MACE group	MACE group	*P*-value
	*n* = 80	*n* = 71	
MVCAD, n (%)	32 (40.0)	42 (59.2)	0.019*
Culprit vessel			0.256
LM, n (%)	0 (0.0)	2 (2.8)	
LAD, n (%)	44 (55.0)	37 (52.1)	
LCx, n (%)	12 (15.0)	6 (8.5)	
RCA, n (%)	24 (30.0)	26 (36.6)	
Post-PCI TIMI flow grade 0 to 2, n (%)	1 (1.3)	3 (4.2)	0.342
LVESD, mm	31.0 (28.0, 34.0)	33.0 (29.0, 37.0)	0.052
LVEDD, mm	46.8 ± 5.3	47.4 ± 5.7	0.516
LVEF, %	60.0 (56.5, 64.0)	58.0 (45.0, 63.0)	0.024*

All data are described as number (percentage), mean ± standard deviation, or median (interquartile range), as appropriate. LAD, left anterior descending artery; LCx, left circumflex artery; LM, left main coronary artery; LVEDD, left ventricular end-diastolic dimension; LVEF, left ventricular ejection fraction; LVESD, left ventricular end-systolic dimension; MVCAD, multivessel coronary artery disease; PCI, percutaneous coronary intervention; RCA, right coronary artery; TIMI, thrombolysis in myocardial infarction

* Statistically significant

### Treatment delays

The differences between the non-MACE and MACE groups in the several treatment delays were not statistically significant (Table 3).

**Table 3:** Treatment delays

Variables	Non-MACE group	MACE group	*P*-value
	*n* = 80	*n* = 71	
S-to-FMC time, mins	110.0 (48.0, 201.8)	83.0 (50.0, 181.0)	0.455
FMC-to-ECG time, mins	2.0 (1.0, 5.0)	2.0 (1.0, 3.0)	0.092
ECG-to-I time, mins	5.0 (3.0, 7.0)	6.0 (3.0, 7.0)	0.864
cTn-to-R time, mins	18.0 (17.0, 18.0)	18.0 (17.0, 18.0)	0.518
Cath lab activation time, mins	15.0 (10.0, 20.0)	15.0 (10.0, 20.0)	0.687
D2B time, mins	65.5 (53.3, 80.0)	69.0 (56.0, 86.0)	0.176
TIT, mins	178.0 (121.0, 281.0)	175.0 (115.0, 286.0)	0.534
D2B time < 90 min, n (%)	71 (88.8)	59 (83.1)	0.316
D2B time < 60 min, n (%)	33 (41.3)	20 (28.2)	0.093

All data are described as number (percentage) or median (interquartile range). Cath lab, catheterization laboratory; cTn-to-R, cardiac troponin-to-result; D2B, door-to-balloon; ECG-to-I, electrocardiogram-to-interpretation; FMC-to-ECG, first medical contact-to-electrocardiogram; S-to-FMC, symptom-to-first medical contact; TIT, total ischemic time

### Univariate and multivariate logistic regression analysis

Logistic correlation analysis was performed with age, SBP, DBP, sCr, cTnI, eGFR, MVCAD, and LVEF for the occurrence of in-hospital MACE (Table 4). Using univariate logistic regression analysis, we found that age, sCr, cTnI, MVCAD, and Killip class III/IV were risk factors for in-hospital MACE after primary PCI in the patients with acute STEMI, whereas eGFR, LVEF, SBP, and DBP were protective factors (P < 0.05). After adjusting for age and other potential confounders, the multivariate logistic regression showed that eGFR, LVEF, cTnI, SBP, and Killip class III/IV were independent predictors of in-hospital MACE after primary PCI in the patients with acute STEMI (P < 0.05).

**Table 4:** Univariate and multivariate logistic regression analysis

Variables	Univariate analysis		Multivariate analysis	
	HR (95% CI)	*P*-value	HR (95% CI)	*P*-value
Age	1.035 (1.010, 1.060)	0.006*		
SBP	0.984 (0.972, 0.996)	0.007*	0.981 (0.967, 0.996)	0.014*
DBP	0.979 (0.962, 9.996)	0.015*		
sCr	5.564 (1.359, 22.779)	0.017*		
eGFR	0.968 (0.950, 0.986)	< 0.001*	0.978 (0.958, 0.999)	0.043*
cTnI	1.019 (1.002, 1.037)	0.033*	1.023 (1.000, 1.045)	0.041*
MVCAD	2.172 (1.133, 4.166)	0.020*		
LVEF	0.933 (0.894, 0.973)	0.001*	0.947 (0.904, 0.992)	0.021*
Killip class III/IV	4.114 (2.005, 8.442)	< 0.001*	2.754 (1.185, 6.400)	0.019*
D2B time < 90 min	1.605 (0.633, 4.069)	0.319		
D2B time < 60 min	1.790 (0.905, 3.542)	0.094		

CI, confidence interval; cTnI: cardiac troponin I; D2B, door-to-balloon; DBP, diastolic blood pressure; eGFR, estimated glomerular filtration rate; LVEF, left ventricular ejection fraction; MVCAD, multivessel coronary artery disease; HR, hazard ratios; SBP, systolic blood pressure; sCr, serum creatinine

* Statistically significant

#### Development, assessment, and validation of the nomogram model

**Fig. 2:**
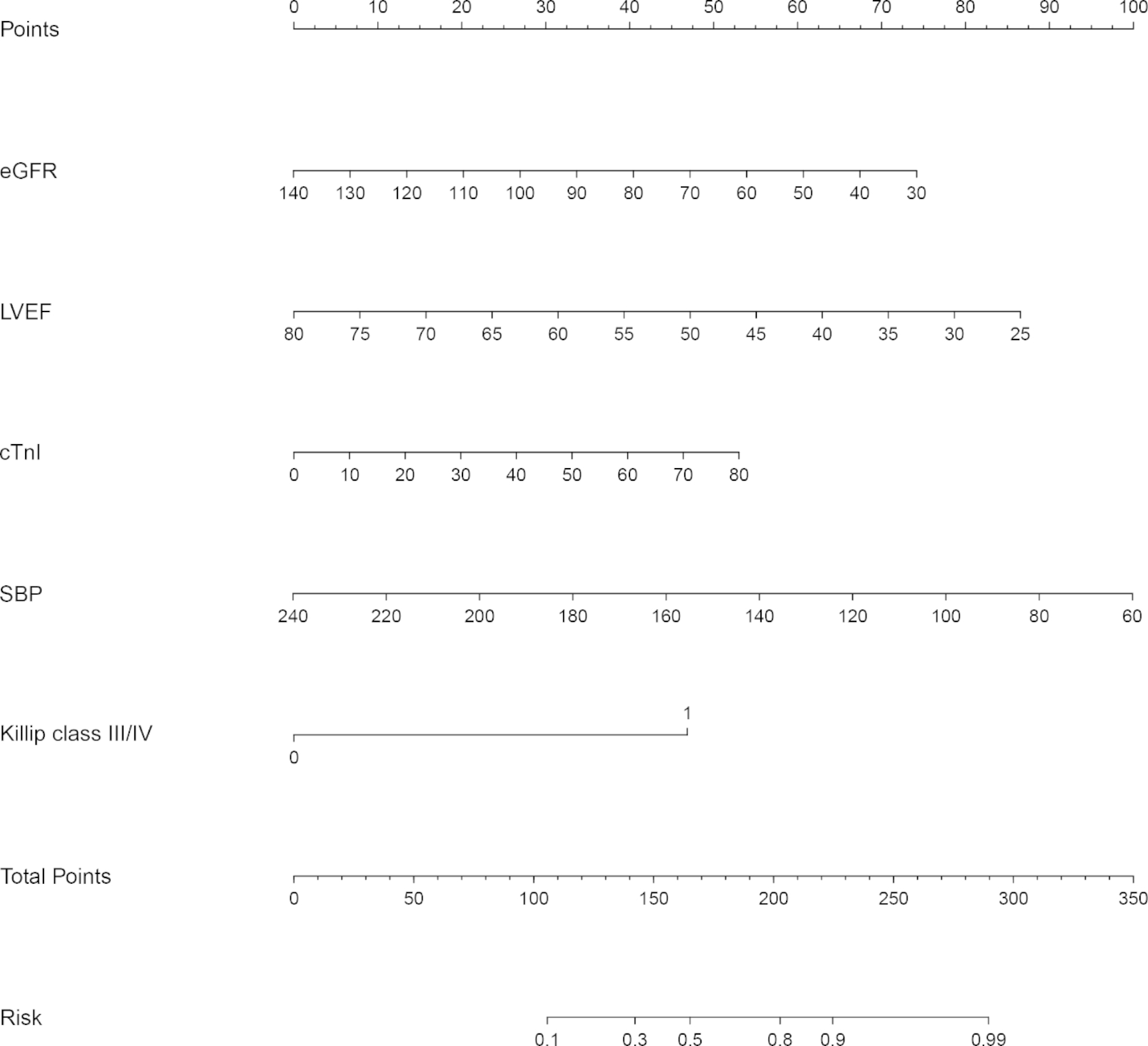
Nomogram for predicting in-hospital MACE. cTnI, cardiac troponin I; eGFR, estimated glomerular filtration rate; LVEF, left ventricular ejection fraction; SBP, systolic blood pressure

A nomogram model was developed based on five independent predictors (eGFR, LVEF, cTnI, SBP, and Killip class III/IV) (Fig. 2). Regarding the weight of variables contained in the model, corresponding points were obtained on the scoring line at the top of nomogram through drawing a vertical line. Finally, the individual probability can be determined on the probability line of in-hospital MACE after primary PCI in patients with acute STEMI through the total points, which can be calculated by adding the points of five factors together.

**Fig. 3:**
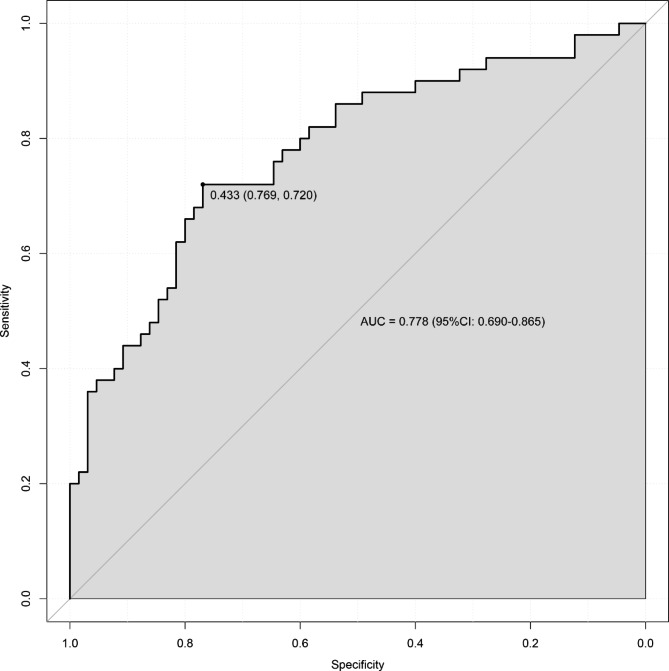
ROC curve for the nomogram. AUC, area under the curve

**Fig. 4:**
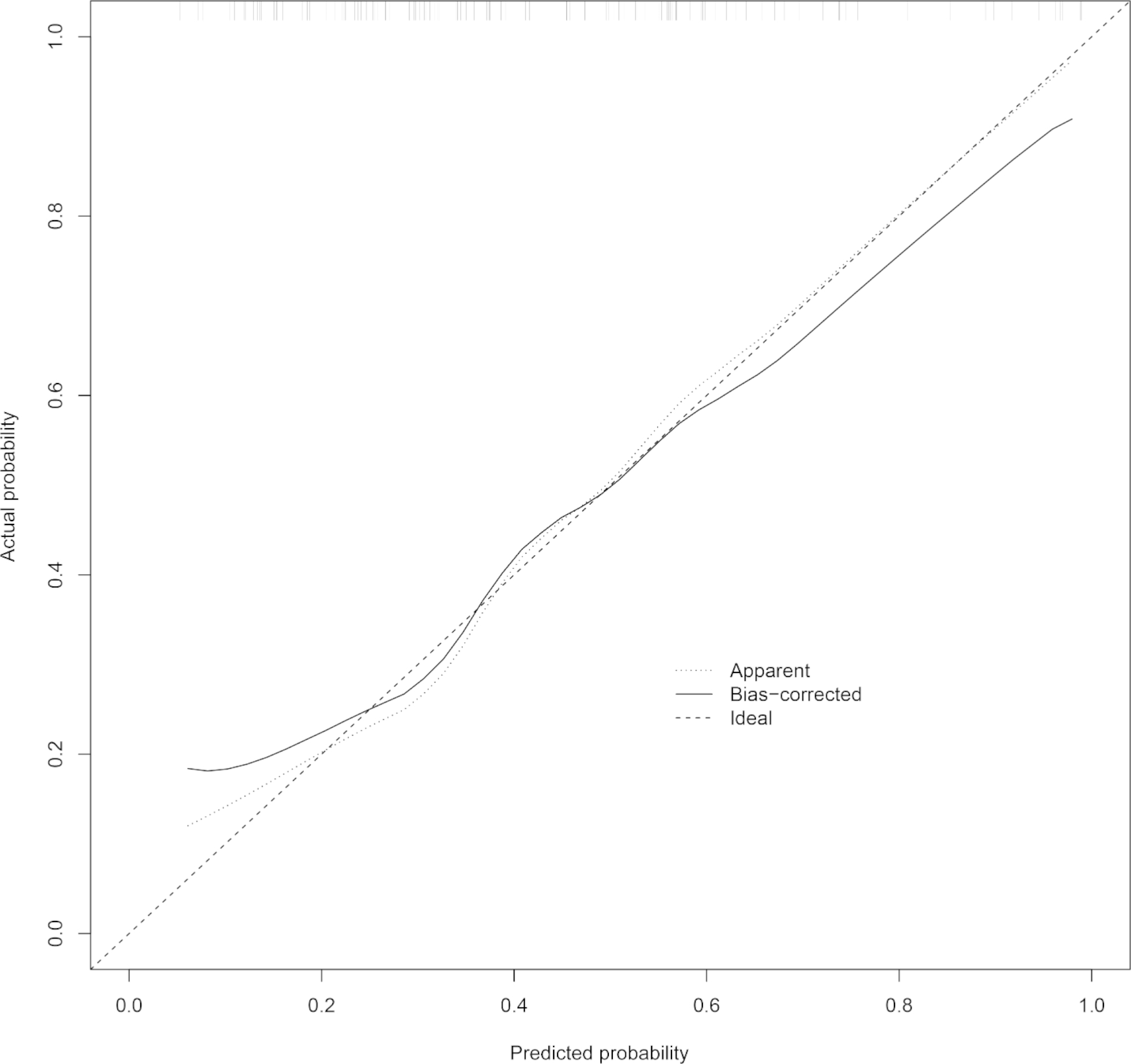
Calibration plot of the nomogram. Ideal line represents perfect prediction that nomogram-predicted probability matches actually observed probability

**Fig. 5:**
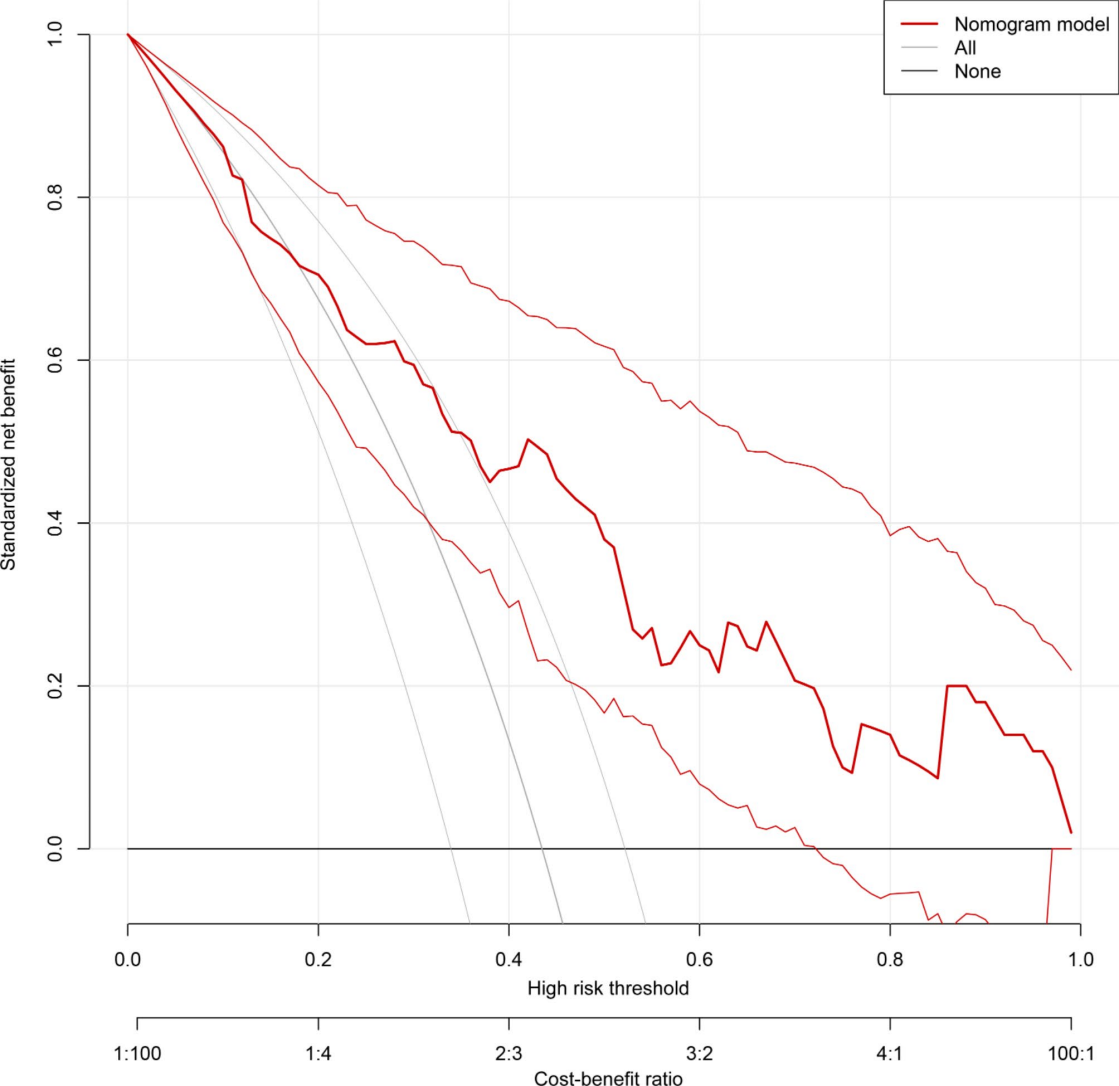
Decision curves of the nomogram. Net benefit is plotted against various probability threshold

Internal validation was performed using bootstrap with 1000 replicates. The receiver operating characteristic curve was drawn to assess the predictive capability of the nomogram (Fig. 3), and the area under the curve was 0. 778 (95%CI: 0.690–0.865), indicating good discrimination of our nomogram. The calibration plot was used to test the goodness-of-fit of the model, which was well calibrated through visual inspection (Fig. 4). The decision curve demonstrated that compared with “intervention for all” or “no intervention” strategies, our predictive model could gain more clinical net benefits with a threshold probability of approximately 20% or greater (Fig. 5).

## Discussion

In our study, the differences in age, SBP, DBP, sCr, eGFR, cTnI, MVCAD, LVEF, and Killip class III/IV between the MACE and non-MACE groups were statistically significant. The present results are consistent with previous reports [9–20]. We found that eGFR, LVEF, cTnI, SBP, and Killip class III/IV independently predicted the occurrence of in-hospital MACE, and the prediction model for in-hospital MACE after primary PCI in patients with acute STEMI had good discrimination, calibration, and clinical utility. To the best of our knowledge, this study is the first clinical study of independent predictors for in-hospital MACE after primary PCI in patients with acute STEMI under the condition that the treatment process meets the quality metrics of the China CPC (standard center).

Recognizing high-risk features is useful for improving the clinical outcomes of patients with acute STEMI because high-risk patients are frequently accompanied by multiple risk factors that can significantly increase the risk of MACE [21]. The association between renal function and cardiovascular disease is well established. Low creatinine excretion is associated with MACE in the general population. For every doubling of creatinine excretion, the risk of MACE is reduced by about 60% in women and by about 25% in men. [12]. According to the Kidney Disease Outcomes Quality Initiative clinical practice guidelines for chronic kidney disease and subsequent guidelines, sCr is not an accurate indicator of glomerular filtration rate (GFR) on its own, and eGFR is considered the ideal indicator for assessing renal function [22, 23]. As GFR decreases, impaired renal function induces oxidative stress and inflammation, leading to endothelial dysfunction and an increased prevalence of metabolic acidosis, which decreases myocardial contractility and the β-adrenergic response [24, 25]. Consistent with earlier studies [13], our study found a negative correlation between baseline eGFR levels and the development of in-hospital MACE. Therefore, in acute STEMI instances involving patients with decreased eGFR or renal insufficiency, cardiologists and nephrologists need to pay more attention.

Severe left ventricular dysfunction caused by STEMI may continue even after effective reperfusion therapy. Routine echocardiography is recommended by the guidelines for all patients with STEMI [8, 26, 27]. LVEF is one of the most examined echocardiographic parameters during admission and follow-up and is a major predictor of MACE in patients with STEMI [17, 18]. The factors contributing to the mechanism of LVEF reduction in patients with STEMI include myocardial compromise due to myocardial necrosis, myocardial stunning, and mechanical complications such as papillary muscle rupture, septal defect, and ventricular free wall rupture [28]. According to a study by Margolis et al., patients with reduced LVEF (below 40%) tended to be older and have more comorbidities, whereas those with mid-range LVEF (40–49%) may progress to heart failure [29]. In our study, LVEF was measured when the patient was stable after primary PCI and independently predicted in-hospital MACE. Furthermore, Son et al. demonstrated that a decreased preprocedural LVEF was associated with an increased risk of MACE after successful primary PCI [30]. Therefore, we believe that monitoring LVEF is of importance in the disease management of patients with acute STEMI.

The assessment of myocardial injury after acute STEMI is crucial for the evaluation of the efficacy of reperfusion therapy and for predicting prognosis [31]. Today, cTn testing is a widely accepted tool for diagnosing AMI, and together with clinical assessment and ECG, it forms the cornerstone of diagnosing patients with acute chest pain. Previous studies have demonstrated that cTnI release directly correlates with the number of necrotic cardiomyocytes and single-point cTnI values provided complementary prognostic information to cardiac magnetic resonance imaging [32]. Post-PCI cTnI is a reliable tool for predicting infarct size [33], and the incidence of MACE is higher in acute STEMI patients with larger infarct sizes after PCI [34]. Our findings support that cTnI provides prognostic information for risk stratification of acute STEMI patients undergoing PCI and can be incorporated into risk prediction models for MACE.

A number of studies have demonstrated an association between a lower incidence of MACE and higher SBP and DBP at admission [10, 11, 19]. In our cohort, both SBP and DBP were lower in the MACE group, and SBP at admission independently predicted in-hospital MACE. Since SBP is a composite of cardiac output and systemic peripheral resistance, SBP levels within the normal range at admission point to more limited necrosis in the myocardial tissue, the absence of severe atrioventricular conduction system disorders, and less pronounced activation of the adrenergic neurohormonal system [35]. In addition, as SBP increases, the blood flow rate may increase, which could reduce the risk of thrombosis [10]. Regularly, SBP and DBP display a highly linear relationship [36]. Since coronary perfusion occurs during diastole, a decrease in DBP may impede coronary blood flow and exacerbate ischemia, especially in arteries where obstruction already exists. The automatic regulation of coronary perfusion occurs naturally; however, coronary artery disease interferes with this intrinsic system. Therefore, a decrease in blood pressure leads to increased ischemia distal to the stenosis [37], and these factors influence whether in-hospital MACE will occur.

The Killip classification is still recommended by social guidelines in the current era as a simple and fast tool for risk stratification in patients with AMI. Patients with a higher Killip class were found to have more severe coronary artery disease, higher incidence of ventricular dysfunction and recurrent ischemia, and larger myocardial infarctions [38]. Consistent with recent studies, our study found that Killip class III/IV was significantly associated with in-hospital MACE [19, 20]. To reduce treatment delays, it is more practical to assess the Killip class at the FMC based on the patient’s signs and symptoms of heart failure and to perform an echocardiogram when the patient was stable after PCI. In this study, Killip class and LVEF reflected the patient’s heart function from different periods and perspectives during the treatment process.

Treatment delay is often considered to be one of the most significant factors in healthcare quality for patients with acute STEMI. In the latest quality metrics and assessment methods for the China CPC (standard center) (https://www.chinacpc.org/home/aview/911), the in-hospital treatment delays contain the following indicators: (a) for all patients with acute chest pain, the mean FMC-to-ECG time per quarter should be within 10 min; (b) for patients with STEMI undergoing primary PCI, the mean D2B time per quarter should be under 90 min and show an improvement trend, and the percentage of D2B time of under 90 min should be no less than 75%. Furthermore, the following requirements are included in the accreditation criteria for the China CPC (standard center) (https://www.chinacpc.org/home/aview/838): (a) the time from sampling to obtaining the results of POCT for cTn is practical, and the mean time does not exceed 20 min; (b) the cath lab activation time is less than 30 min and shows a tendency to shorten or stabilize; (c) within 10 min of the performance of the first ECG, it is interpreted by a diagnostically competent physician. According to the requirements of the China CPC accreditation standards, the first accreditation has a three-year validity duration. Reaccreditation is valid for an additional five years, with reaccreditations once every five years. Moreover, the reaccreditation criteria for the China CPC (standard center) (https://www.chinacpc.org/home/aview/359) further suggest that there should be a steady-state trend when the monthly mean D2B time is under 60 min. This indicates that although the current assessment criterion is still 90 min, the headquarter of the China CPC believes that a more appropriate target for D2B time is to consistently be 60 min or less. These quality metrics focus on the D2B time and its components. In our study, all treatment procedures met the accreditation criteria and quality metrics of the China CPC (standard center). It may appear that treatment delay was unrelated to the occurrence of in-hospital MACE because the distribution of each treatment delay was not significantly different between the non-MACE and MACE groups. However, due to the differences in the prognosis of patients in the overall cohort, this situation can be explained from another perspective: after treatment delays met the accreditation criteria and quality metrics of the China CPC (standard center), whether patients experienced in-hospital MACE was related to their condition but not to treatment delays. As in the study by Nallamothu et al., D2B time was consistently linked to decreased mortality at the population level, and the long-term trend of increasing mortality was largely attributable to the increased proportion of high-risk patients undergoing primary PCI [39]. This suggests that the current accreditation criteria and quality metrics of the China CPC (standard center) are relatively reasonable in terms of treatment delays. Although we lack records of treatment delays before CPC accreditation to compare the impact of treatment delays on in-hospital MACE occurrence before and after CPC accreditation, multiple previous studies have demonstrated that China CPC accreditation is associated with improved in-hospital clinical outcomes and better management for patients with STEMI, including a lower risk of MACE and in-hospital mortality and more PCI use [6, 40].

The current guidelines from the American College of Cardiology and the American Heart Association recommend that the D2B time should be no more than 90 min [26], and this is a valid predictor of patients for the evaluation of treatment outcomes. This standard has also been embraced by the China CPC. Early studies associated a shorter patient-specific D2B time with lower mortality after PCI at the individual level [41, 42], but whether a shorter D2B time is associated with MACE has been controversial [43]. In our study, approximately 90% of the patients had a procedure that met the CPC requirement for the D2B time to be 90 min or less, and its distribution was not significantly different between the two groups. Only approximately 35% of the patients included in this study had a D2B time of fewer than 60 min, but a further reduction of the D2B time to 60 min or less was not indicated to be associated with a lower risk of in-hospital MACE. Previous studies have pointed out that the relationship between time-to-treatment and outcomes in STEMI is nonlinear, with the benefit of reperfusion decreasing over time [39]; lowering D2B time (90 min or less) has been proven to be beneficial in reducing mortality in patients with STEMI, but a further decrease in D2B time may not further optimize the outcomes [44–46]. Additionally, attempting to shorten the D2B time may have the unintended consequence of increasing the number of unnecessary PCI, where patients are transported urgently to the cardiac cath lab even though there is no STEMI present [44]. These patients misdiagnosed with STEMI would subsequently be at higher risk for PCI and delayed diagnosis and treatment of their actual urgent issues. In the “2013 ACCF/AHA Guideline for the Management of ST-Elevation Myocardial Infarction”, the FMC-to-device time was introduced while the D2B time was retained, which means the starting point for evaluating treatment delays was moved forward from arriving at the hospital door to the FMC, and more emphasis was placed on planning and requiring the entire emergency treatment system as a whole [26]. In addition, in the “2017 ESC Guidelines for the management of acute myocardial infarction in patients presenting with ST-segment elevation”, the D2B time has been removed as a vague term [27]. Moreover, the “2019 Chinese Society of Cardiology (CSC) guidelines for the diagnosis and management of patients with ST-segment elevation myocardial infarction” also recommend that the management of patients with STEMI begins with FMC, thereby maximizing reperfusion efficiency [8]. However, certain standards-setting compromises had to be made in light of China’s circumstances and characteristics. In fact, due to the uneven distribution of healthcare resources across regions, the local governments and public healthcare systems in China seek to develop the healthcare sector in the regions they manage, thus favoring the construction of large medical institutions to reduce the outflow of patients, while primary care facilities are underequipped and low-skilled. From a treatment process perspective, the outpatient department of large medical institutions corresponds to primary care in Western countries, and the inpatient department corresponds to secondary care and tertiary care. They belong to the same hospital and offer seamless one-stop treatment. These factors lead patients to ignore primary care facilities and go directly to large medical institutions. A hierarchical medical system has been advanced in China’s medical system for many years. However, for a long time, primary care cannot be implemented, and the two-way referral mechanism is not ideal, which together form a state of graded but not triaged management. In addition, due to the late start of China’s prehospital EMS construction, the unreasonable layout of the emergency network and the severe shortage of prehospital emergency personnel, patients often choose to go to hospitals on their own instead of calling EMS. Although the basic concept of China CPC accreditation is to build a regional collaborative treatment system centered on hospitals where PCI can be performed for AMI patients, the current accreditation system is only for individual medical institutions. While these large medical institutions certified as standard centers can effectively reduce the D2B time, they have had limited improvement in the S-to-FMC time and FMC-to-device time. Therefore, it is more practical to make the D2B time instead of the FMC-to-device time and TIT as the assessment target for the accreditation of the China CPC (standard center), and it is worthwhile to continue to exist. However, continued efforts on reducing the D2B time to 60 min or less for use as a quality metric and performance indicator for the China CPC (standard center) must consider both the possible benefits and unanticipated repercussions of such actions. This deserves continued observation in future practice to elucidate the true impact of further reducing D2B time on patient outcomes.

The currently accepted measures of healthcare quality in the treatment of STEMI focus on shortening in-hospital treatment delays. However, this concern does not take into consideration how long myocardial ischemia lasts before the patients go to the hospital. Both animal experiments and clinical studies have shown that the shorter the TIT is, the smaller the infarct size, the less microvascular obstruction, and the more myocardium that can be saved [47, 48]. Recent studies have shown that TIT is an independent risk factor for MACE and may be a better indicator of the extent of myocardial injury and necrosis than D2B [49, 50]. Meisel et al. proposed a pathophysiologic mechanism that seems to provide a plausible explanation for the current findings. If myocardial ischemia is not rectified, myocardial necrosis develops, initially causing rapid damage, and the progression of occlusion slows with time. In the lack of immediate treatment, the period featuring a sharp increase in the myonecrosis rate seems to occur typically within TIT, while the D2B time appears later during the flat slope of the time-myonecrosis curve. Therefore, a shorter D2B time usually does not affect the long-term prognosis of patients with STEMI, whereas a longer TIT does [51]. Within two to three hours of symptom onset, the impact of reperfusion on myocardial rescue is significantly reduced; despite a D2B time of 90 min or less, patients with TIT longer than 180 min have a worse prognosis [52, 53]. Unfortunately, only approximately 40% of the patients included in this study had a TIT of fewer than three hours, which indicates that by the time patients arrived at the hospital, more than half had already missed the window of time in which reperfusion could have a maximum effect. In addition, the hospital where the study was conducted is located in a large city in China with a high density of large medical institutions, allowing patients to choose one in their vicinity. Thus, the patients included in this study primarily lived in urban areas or suburbs with convenient transportation, and they were infrequently kept waiting for long once they decided to seek medical care. In this case, the S-to-FMC time was not excessively prolonged, and the D2B time could be controlled to be stable by the standardized treatment process, resulting in a relatively concentrated distribution of TIT. This may help to explain why there is no significant difference in TIT between the two groups. As the D2B time decreases, it becomes a diminishing proportion of TIT, and its relative importance decreases as TIT increases. However, in China, there are no special regulations other than the D2B time and its components. Therefore, we should increase the awareness of TIT, which may be the proper focus of attention for acute STEMI treatment. Even so, it is unrealistic to use TIT as a quality metric for the China CPC (standard center). As mentioned earlier, a typical problem at present is that the CPC accreditation of standard centers can only optimize the in-hospital emergency processes for large medical institutions. In response, the China Alliance of CPC is establishing chest pain treatment units for primary care facilities (township health centers, community medical service centers, etc.), along with the preliminary center accreditation, to form a regional collaborative treatment system by integrating local medical resources. Shortening the TIT may be a more appropriate goal for regional collaborative treatment system quality improvement programs.

The prolonged time it takes for patients to recognize ischemic symptoms may be the main reason for the long TIT, which makes the S-to-FMC time an important factor. Our society may be aware of typical AMI symptoms, but many still lack awareness of possible atypical presentations, such as an upset stomach, which are reported to be significant predictors of delay before hospitalization [54]. Compared to the median S-to-FMC time of two to three hours in Western countries, the S-to-FMC time is still longer (3.3 to 3.5 h) in China [55], and over half of the patients are transferred to the hospital by private vehicles rather than ambulances, with the former having a longer S-to-FMC time [56]. In addition, the prolongation of S-to-FMC time not only directly increases the TIT but also indirectly affects the TIT by predicting the D2B time; factors such as the awareness of patients and families about the disease and their financial status can influence the length of time it takes to generate a willingness to seek medical care and sign informed consent. In our study, the S-to-FMC time was a major component of TIT (approximately 60% on the mean), and the proportion of patients who came to the hospital without calling EMS was close to 90%. These patients did not receive the benefit of antiplatelet and antithrombotic therapy during the S-to-FMC time. The prognosis of patients with acute STEMI can be further improved by minimizing the S-to-FMC time. For instance, by raising public knowledge of the early AMI signs and symptoms, EMS can be activated more quickly for an early diagnosis, and PCI can be performed for the best prognosis. With the widespread establishment of the China CPC regional collaborative treatment system, we can anticipate that the cooperation between relevant individuals and groups, such as patients, healthcare professionals, hospitals, and emergency centers, can produce positive results to achieve the mutual goal of focusing on improving the way healthcare is delivered and provide a model for the current and future of the healthcare industry.

Certain limitations should be taken into account when interpreting the results of the current study. First, this was a retrospective study. Despite statistical adjustments, the presence of unmeasured confounders could not be avoided. The eGFR was calculated retrospectively from a single measurement of the sCr level and is thereby influenced by the choice of the calculation method. In addition, the sample size of this study is small and may be partially distorted by sampling error. Although this study is based on data from a well-documented CPC registry, this study is only a single-center study. Patient samples from other hospitals were not included, and the experiences of other hospitals may vary. Next, when we tried to focus on treatment delays that are more directly controlled by hospitals, we lacked precise documentation of the time of symptom onset in some patients, an essential aspect of TIT, but it was limited by the patients’ and their families’ perceptions of AMI and was inevitably subject to recall bias. China is a country with significant regional and hospital variation, and with increasing patient complexity and disparities in treatment, it is clear that our findings are not representative of general practice in China. Finally, important information such as long-term outcomes and events, SYNTAX scores, and PCI methods were not assessed in this study. This should be validated in a larger cohort to confirm the prognosis of patients.

## Conclusion

Our data suggest that eGFR, LVEF, cTnI, SBP, and Killip class III/IV independently predict in-hospital MACE after primary PCI in patients with acute STEMI, and the prediction model constructed based on the above factors could be useful for individual risk assessment and early management guidance.

## Data Availability

The data that support the findings of this study are available on request from the corresponding author after the ethics committee’s agreement. The data are not publicly available due to their containing information that could compromise the privacy of patients.

## Abbreviations

AMI: Acute myocardial infarction
AUC: Area under the curve
Cath lab: Catheterization laboratory
CI: Confidence interval
CPC: Chest pain center
cTn: Cardiac troponin
cTnI: Cardiac troponin I
cTn-to-R: Cardiac troponin-to-result
D2B: Door-to-balloon
DBP: Diastolic blood pressure
ECG: Electrocardiogram
ECG-to-I: Electrocardiogram-to-interpretation
eGFR: Estimated glomerular filtration rate
EMS: Emergency medical services
FMC: First medical contact
FMC-to-ECG: First medical contact-to-electrocardiogram
HDL-C: High-density lipoprotein cholesterol
HR: Hazard ratios
LAD: Left anterior descending artery
LCx: Left circumflex artery
LDL-C: Low-density lipoprotein cholesterol
LM: Left main coronary artery
LVEDD: Left ventricular end-diastolic dimension
LVEF: Left ventricular ejection fraction
LVESD: Left ventricular end-systolic dimension
MACE: Major adverse cardiovascular events
MVCAD: Multivessel coronary artery disease
PCI: Percutaneous coronary intervention
POCT: Point-of-care testing
RCA: Right coronary artery
ROC: Receiver operating characteristic
SBP: Systolic blood pressure
sCr: Serum creatinine
STEMI: ST-segment elevation myocardial infarction
TIMI: Thrombolysis in myocardial infarction
TIT: Total ischemic time

## References

[1] Reed GW, Rossi JE, Cannon CP (2017). Acute myocardial infarction. Lancet.

[2] Puymirat E, Simon T, Cayla G, Cottin Y, Elbaz M, Coste P, Lemesle G, Motreff P, Popovic B, Khalife K (2017). Acute myocardial infarction: changes in patient characteristics, management, and 6-Month Outcomes over a period of 20 years in the FAST-MI program (French Registry of Acute ST-Elevation or Non-ST-Elevation myocardial infarction) 1995 to 2015. Circulation.

[3] Jang SJ, Yeo I, Feldman DN, Cheung JW, Minutello RM, Singh HS, Bergman G, Wong SC, Kim LK (2020). Associations between Hospital length of Stay, 30-Day readmission, and costs in ST-Segment-Elevation myocardial infarction after primary percutaneous coronary intervention: a Nationwide Readmissions Database Analysis. J Am Heart Assoc.

[4] Zhou M, Wang H, Zeng X, Yin P, Zhu J, Chen W, Li X, Wang L, Wang L, Liu Y (2019). Mortality, morbidity, and risk factors in China and its provinces, 1990–2017: a systematic analysis for the global burden of Disease Study 2017. Lancet.

[5] Li J, Li X, Wang Q, Hu S, Wang Y, Masoudi FA, Spertus JA, Krumholz HM, Jiang L (2015). ST-segment elevation myocardial infarction in China from 2001 to 2011 (the China PEACE-Retrospective Acute Myocardial Infarction Study): a retrospective analysis of hospital data. Lancet.

[6] Fan F, Li Y, Zhang Y, Li J, Liu J, Hao Y, Smith SC, Fonarow GC, Taubert KA, Ge J (2019). Chest Pain Center Accreditation is Associated with Improved In-Hospital outcomes of Acute myocardial infarction patients in China: findings from the CCC-ACS project. J Am Heart Assoc.

[7] Tsukui T, Sakakura K, Taniguchi Y, Yamamoto K, Seguchi M, Jinnouchi H, Wada H, Fujita H (2020). Factors associated with poor clinical outcomes of ST-elevation myocardial infarction in patients with door-to-balloon time < 90 minutes. PLoS ONE.

[8] Chinese Society of Cardiology of Chinese Medical Association (2019). Editorial Board of Chinese Journal of Cardiology: 2019 chinese society of Cardiology (CSC) guidelines for the diagnosis and management of patients with ST-segment elevation myocardial infarction. Chin J Cardiol.

[9] Gong X, Zhou L, Dong T, Ding X, Zhao H, Chen H, Li H (2022). Impact of COVID-19 pandemic on STEMI undergoing primary PCI treatment in Beijing, China. Am J Emerg Med.

[10] Pei J, Wang X, Xing Z, Chen P, Su W, Deng S, Hu X (2020). Association between admission systolic blood pressure and major adverse cardiovascular events in patients with acute myocardial infarction. PLoS ONE.

[11] Liu YH, Dai YN, Wang LT, Chen PY, Zeng LH, Zhang YS, Duan CY, Chen JY, Tan N, He PC (2022). Association between DBP and major adverse cardiovascular events in patients with ST-segment elevation myocardial infarction undergoing percutaneous coronary intervention. J Hypertens.

[12] Oterdoom LH, Gansevoort RT, Schouten JP, de Jong PE, Gans RO, Bakker SJ (2009). Urinary creatinine excretion, an indirect measure of muscle mass, is an independent predictor of cardiovascular disease and mortality in the general population. Atherosclerosis.

[13] Go AS, Chertow GM, Fan D, McCulloch CE, Hsu CY (2004). Chronic kidney disease and the risks of death, cardiovascular events, and hospitalization. N Engl J Med.

[14] Sorajja P, Gersh BJ, Cox DA, McLaughlin MG, Zimetbaum P, Costantini C, Stuckey T, Tcheng JE, Mehran R, Lansky AJ (2007). Impact of multivessel disease on reperfusion success and clinical outcomes in patients undergoing primary percutaneous coronary intervention for acute myocardial infarction. Eur Heart J.

[15] Zhao X, Wang Y, Liu C, Zhou P, Sheng Z, Li J, Zhou J, Chen R, Chen Y, Zhao H et al. Association between Variation of Troponin and Prognosis of Acute Myocardial Infarction before and after Primary Percutaneous Coronary Intervention. *J Interv Cardiol* 2020, 2020:4793178. 10.1155/2020/4793178 PMC7399759 32774185

[16] Ryu KS, Park HW, Park SH, Shon HS, Ryu KH, Lee DG, Bashir ME, Lee JH, Kim SM, Lee SY (2015). Comparison of clinical outcomes between culprit vessel only and multivessel percutaneous coronary intervention for ST-segment elevation myocardial infarction patients with multivessel coronary diseases. J Geriatr Cardiol.

[17] Casas G, Limeres J, Oristrell G, Gutierrez-Garcia L, Andreini D, Borregan M, Larrañaga-Moreira JM, Lopez-Sainz A, Codina-Solà M, Teixido-Tura G (2021). Clinical risk prediction in patients with left ventricular myocardial noncompaction. J Am Coll Cardiol.

[18] Pan D, Xiao S, Hu Y, Pan Q, Wu Q, Wang X, Liu Q, Liu A, Liu J, Zhu H et al. Clinical Nomogram to Predict Major Adverse Cardiac Events in Acute Myocardial Infarction Patients within 1 Year of Percutaneous Coronary Intervention. *Cardiovasc Ther* 2021, 2021:3758320. 10.1155/2021/3758320 PMC8687843 34987604

[19] Wu C, Huo X, Liu J, Zhang L, Bai X, Hu S, Li X, Lu J, Zheng X, Li J (2021). Development and validation of a risk prediction model for in-hospital major cardiovascular events in patients hospitalised for acute myocardial infarction. BMJ Open.

[20] Oh S, Jeong MH, Cho KH, Kim MC, Sim DS, Hong YJ, Kim JH, Ahn Y. Outcomes of Nonagenarians with Acute Myocardial Infarction with or without Coronary Intervention. J Clin Med 2022, 11(6). 10.3390/jcm11061593 PMC8955178 35329920

[21] Cesaro A, Gragnano F, Calabrò P, Moscarella E, Santelli F, Fimiani F, Patti G, Cavallari I, Antonucci E, Cirillo P (2021). Prevalence and clinical implications of eligibility criteria for prolonged dual antithrombotic therapy in patients with PEGASUS and COMPASS phenotypes: insights from the START-ANTIPLATELET registry. Int J Cardiol.

[22] National Kidney Foundation (2002). K/DOQI clinical practice guidelines for chronic kidney disease: evaluation, classification, and stratification. Am J Kidney Dis.

[23] Levey AS, Coresh J, Balk E, Kausz AT, Levin A, Steffes MW, Hogg RJ, Perrone RD, Lau J, Eknoyan G (2003). National kidney Foundation practice guidelines for chronic kidney disease: evaluation, classification, and stratification. Ann Intern Med.

[24] Yilmaz MI, Stenvinkel P, Sonmez A, Saglam M, Yaman H, Kilic S, Eyileten T, Caglar K, Oguz Y, Vural A (2011). Vascular health, systemic inflammation and progressive reduction in kidney function; clinical determinants and impact on cardiovascular outcomes. Nephrol Dial Transplant.

[25] Schotola H, Toischer K, Popov AF, Renner A, Schmitto JD, Gummert J, Quintel M, Bauer M, Maier LS, Sossalla S (2012). Mild metabolic acidosis impairs the β-adrenergic response in isolated human failing myocardium. Crit Care.

[26] O’Gara PT, Kushner FG, Ascheim DD, Casey DE, Chung MK, de Lemos JA, Ettinger SM, Fang JC, Fesmire FM, Franklin BA (2013). 2013 ACCF/AHA guideline for the management of ST-elevation myocardial infarction: a report of the American College of Cardiology Foundation/American Heart Association Task Force on Practice Guidelines. Circulation.

[27] Ibanez B, James S, Agewall S, Antunes MJ, Bucciarelli-Ducci C, Bueno H, Caforio ALP, Crea F, Goudevenos JA, Halvorsen S (2018). 2017 ESC Guidelines for the management of acute myocardial infarction in patients presenting with ST-segment elevation: the Task Force for the management of acute myocardial infarction in patients presenting with ST-segment elevation of the European Society of Cardiology (ESC). Eur Heart J.

[28] Jenča D, Melenovský V, Stehlik J, Staněk V, Kettner J, Kautzner J, Adámková V, Wohlfahrt P (2021). Heart failure after myocardial infarction: incidence and predictors. ESC Heart Fail.

[29] Margolis G, Khoury S, Ben-Shoshan J, Letourneau-Shesaf S, Flint N, Keren G, Shacham Y (2017). Prognostic implications of Mid-Range Left Ventricular Ejection Fraction on Patients presenting with ST-Segment Elevation myocardial infarction. Am J Cardiol.

[30] Son YJ, Shim SK, Hwang SY, Ahn JH, Yu HY (2016). Impact of left ventricular ejection fraction and medication adherence on major adverse cardiac events during the first year after successful primary percutaneous coronary interventions. J Clin Nurs.

[31] Halkin A, Stone GW, Grines CL, Cox DA, Rutherford BD, Esente P, Meils CM, Albertsson P, Farah A, Tcheng JE (2006). Prognostic implications of creatine kinase elevation after primary percutaneous coronary intervention for acute myocardial infarction. J Am Coll Cardiol.

[32] Ohlmann P, Jaquemin L, Morel O, El Behlgiti R, Faure A, Michotey MO, Beranger N, Roul G, Schneider F, Bareiss P (2006). Prognostic value of C-reactive protein and cardiac troponin I in primary percutaneous interventions for ST-elevation myocardial infarction. Am Heart J.

[33] Hallén J, Buser P, Schwitter J, Petzelbauer P, Geudelin B, Fagerland MW, Jaffe AS, Atar D (2009). Relation of cardiac troponin I measurements at 24 and 48 hours to magnetic resonance-determined infarct size in patients with ST-elevation myocardial infarction. Am J Cardiol.

[34] Reindl M, Holzknecht M, Tiller C, Lechner I, Schiestl M, Simma F, Pamminger M, Henninger B, Mayr A, Klug G (2020). Impact of infarct location and size on clinical outcome after ST-elevation myocardial infarction treated by primary percutaneous coronary intervention. Int J Cardiol.

[35] Pitsavos C, Panagiotakos D, Zombolos S, Mantas Y, Antonoulas A, Stravopodis P, Kogias Y, Kourlaba G, Tsiamis E, Stefanadis C (2008). Systolic blood pressure on admission predicts in-hospital mortality among patients presenting with acute coronary syndromes: the greek study of acute coronary syndromes. J Clin Hypertens (Greenwich).

[36] Gavish B, Ben-Dov IZ, Bursztyn M (2008). Linear relationship between systolic and diastolic blood pressure monitored over 24 h: assessment and correlates. J Hypertens.

[37] Warren J, Nanayakkara S, Andrianopoulos N, Brennan A, Dinh D, Yudi M, Clark D, Ajani AE, Reid CM, Selkrig L (2019). Impact of Pre-Procedural Blood pressure on long-term outcomes following percutaneous coronary intervention. J Am Coll Cardiol.

[38] El-Menyar A, Zubaid M, AlMahmeed W, Sulaiman K, AlNabti A, Singh R, Al Suwaidi J (2012). Killip classification in patients with acute coronary syndrome: insight from a multicenter registry. Am J Emerg Med.

[39] Nallamothu BK, Normand SL, Wang Y, Hofer TP, Brush JE, Messenger JC, Bradley EH, Rumsfeld JS, Krumholz HM (2015). Relation between door-to-balloon times and mortality after primary percutaneous coronary intervention over time: a retrospective study. Lancet.

[40] Sun P, Li J, Fang W, Su X, Yu B, Wang Y, Li C, Chen H, Wang X, Zhang B (2021). Effectiveness of chest pain centre accreditation on the management of acute coronary syndrome: a retrospective study using a national database. BMJ Qual Saf.

[41] Lambert L, Brown K, Segal E, Brophy J, Rodes-Cabau J, Bogaty P (2010). Association between timeliness of reperfusion therapy and clinical outcomes in ST-elevation myocardial infarction. JAMA.

[42] Rathore SS, Curtis JP, Chen J, Wang Y, Nallamothu BK, Epstein AJ, Krumholz HM (2009). Association of door-to-balloon time and mortality in patients admitted to hospital with ST elevation myocardial infarction: national cohort study. BMJ.

[43] Brennan AL, Andrianopoulos N, Duffy SJ, Reid CM, Clark DJ, Loane P, New G, Black A, Yan BP, Brooks M (2014). Trends in door-to-balloon time and outcomes following primary percutaneous coronary intervention for ST-elevation myocardial infarction: an australian perspective. Intern Med J.

[44] Askandar S, Bob-Manuel T, Singh P, Khouzam RN (2017). Shorter Door-To-Balloon ST-Elevation myocardial infarction time: should there be a Minimum Limit?. Curr Probl Cardiol.

[45] Sutton NR, Gurm HS (2015). Door to Balloon Time: is there a point that is too short?. Prog Cardiovasc Dis.

[46] Menees DS, Peterson ED, Wang Y, Curtis JP, Messenger JC, Rumsfeld JS, Gurm HS (2013). Door-to-balloon time and mortality among patients undergoing primary PCI. N Engl J Med.

[47] Reimer KA, Lowe JE, Rasmussen MM, Jennings RB (1977). The wavefront phenomenon of ischemic cell death. 1. Myocardial infarct size vs duration of coronary occlusion in dogs. Circulation.

[48] Francone M, Bucciarelli-Ducci C, Carbone I, Canali E, Scardala R, Calabrese FA, Sardella G, Mancone M, Catalano C, Fedele F (2009). Impact of primary coronary angioplasty delay on myocardial salvage, infarct size, and microvascular damage in patients with ST-segment elevation myocardial infarction: insight from cardiovascular magnetic resonance. J Am Coll Cardiol.

[49] Chandrasekhar J, Marley P, Allada C, McGill D, O’Connor S, Rahman M, Tan R, Hosseiny AD, Shadbolt B, Farshid A (2017). Symptom-to-balloon time is a strong predictor of adverse events following primary percutaneous coronary intervention: results from the australian Capital Territory PCI Registry. Heart Lung Circ.

[50] Redfors B, Mohebi R, Giustino G, Chen S, Selker HP, Thiele H, Patel MR, Udelson JE, Ohman EM, Eitel I (2021). Time Delay, Infarct size, and microvascular obstruction after primary percutaneous coronary intervention for ST-Segment-Elevation myocardial infarction. Circ Cardiovasc Interv.

[51] Meisel SR, Kleiner-Shochat M, Abu-Fanne R, Frimerman A, Danon A, Minha S, Levi Y, Blatt A, Mohsen J, Shotan A (2021). Direct admission of patients with ST-Segment-Elevation myocardial infarction to the catheterization Laboratory Shortens Pain-to-balloon and door-to-balloon time intervals but only the Pain-to-balloon interval impacts short- and long-term mortality. J Am Heart Assoc.

[52] Shiomi H, Nakagawa Y, Morimoto T, Furukawa Y, Nakano A, Shirai S, Taniguchi R, Yamaji K, Nagao K, Suyama T (2012). Association of onset to balloon and door to balloon time with long term clinical outcome in patients with ST elevation acute myocardial infarction having primary percutaneous coronary intervention: observational study. BMJ.

[53] Brodie BR, Gersh BJ, Stuckey T, Witzenbichler B, Guagliumi G, Peruga JZ, Dudek D, Grines CL, Cox D, Parise H (2010). When is door-to-balloon time critical? Analysis from the HORIZONS-AMI (Harmonizing Outcomes with revascularization and stents in Acute myocardial infarction) and CADILLAC (controlled Abciximab and device investigation to Lower Late Angioplasty Complications) trials. J Am Coll Cardiol.

[54] McKee G, Mooney M, O’Donnell S, O’Brien F, Biddle MJ, Moser DK (2013). Multivariate analysis of predictors of pre-hospital delay in acute coronary syndrome. Int J Cardiol.

[55] Wang L, Zhou Y, Qian C, Wang Y (2017). Clinical characteristics and improvement of the guideline-based management of acute myocardial infarction in China: a national retrospective analysis. Oncotarget.

[56] Peng YG, Feng JJ, Guo LF, Li N, Liu WH, Li GJ, Hao G, Zu XL (2014). Factors associated with prehospital delay in patients with ST-segment elevation acute myocardial infarction in China. Am J Emerg Med.

